# Supplementary material for: Three-Dimensional Tumor Spheroids as a Tool for Reliable Investigation of Combined Gold Nanoparticle and Docetaxel Treatment
Source: Cancers (Basel). 2021 Mar 23;13(6):1465. doi: 10.3390/cancers13061465 (PMC8004664; doi:10.3390/cancers13061465)
Supplement: Supplementary file 1 [file cancers-13-01465-s001.pdf]

cancers

Supplementary Materials

Three-Dimensional Tumor Spheroids as a Tool for Reliable Investigation of Combined Gold Nanoparticle and Docetaxel Treatment

Kyle Bromma, Abdulaziz Alhussan, Monica Mesa Perez, Perry Howard, Wayne Beckham and Devika B. Chithrani

a

Sample	Hydrodynamic Diameter [nm]	Polydispersity Index [%]	Zeta Potential [mV]
15 nm	19.77	11.27	-43.70 ± 6.16
15 nm + PEG	27.82	22.61	-20.07 ± 0.93
15 nm + PEG + RGD	31.00	19.91	-1.09 ± 0.27
50 nm	53.83	27.34	-38.12 ± 1.26
50 nm + PEG	56.66	25.59	-27.75 ± 1.38
50 nm + PEG + RGD	59.20	24.33	10.30 ± 0.51

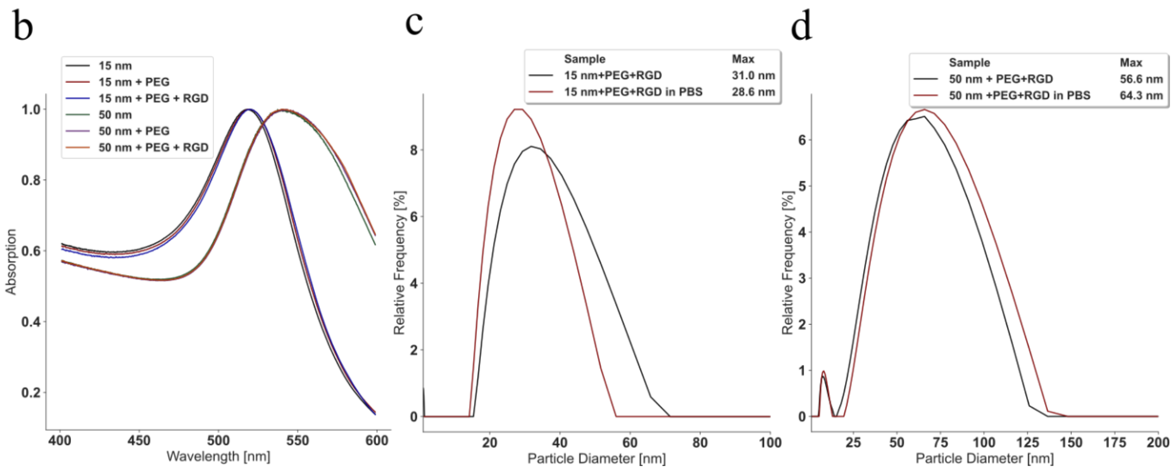

Figure S1. Gold Nanoparticle Characterization. (a) Measured quantities of the GNPs using dynamic light scattering and zeta potential. (b) UV-Vis measurements of the GNPs after each conjugation step, ensuring stability. (c-d) Dynamic light scattering measurements of the GNPs in the presence of phosphate buffered saline (PBS), to ensure stability in serum.

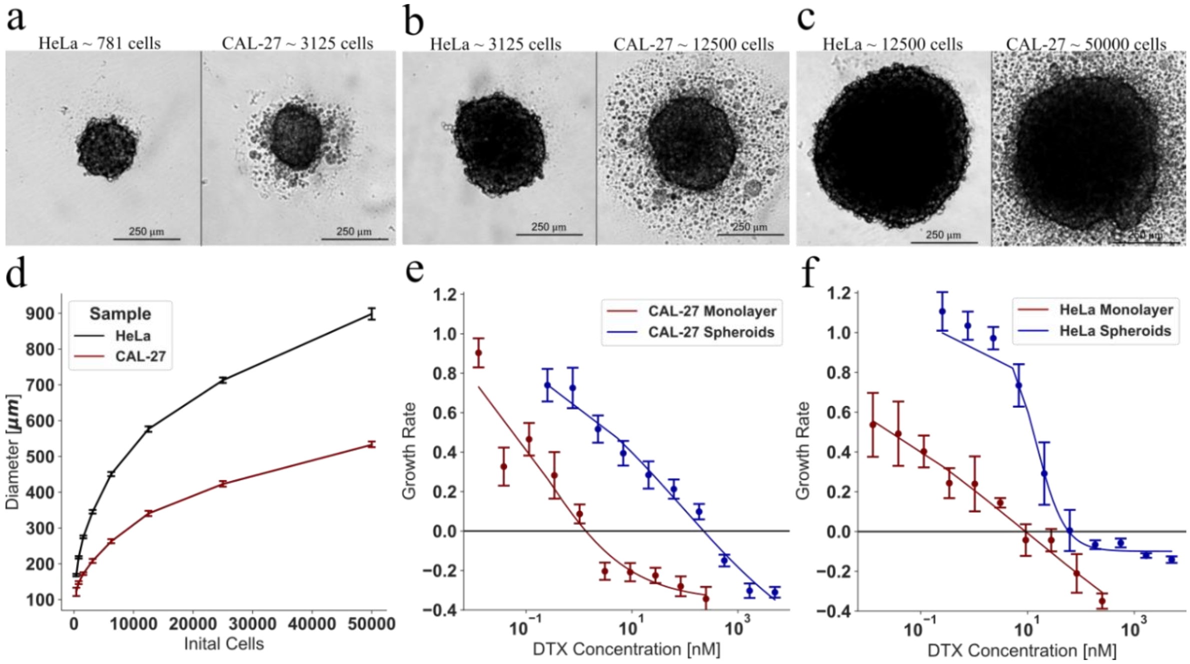

Figure S2. Spheroid Characterization. (a-c) The size of the spheroids under different initial cell counts was approximated using brightfield images taken after three days of incubation. (d) Size of spheroid for the two different cell lines under different initial cell count conditions. An approximate size range of 300 µm - 400 µm was utilized for all experiments. (e-f) Proliferation assay results after treatment of docetaxel of both (e) CAL-27 and (f) HeLa when in monolayer and spheroid. Points are average of three plates of eight samples, line is the best fit using Growth Rate Inhibition as defined by Hafner et al. Error bars are one standard deviation from the mean.

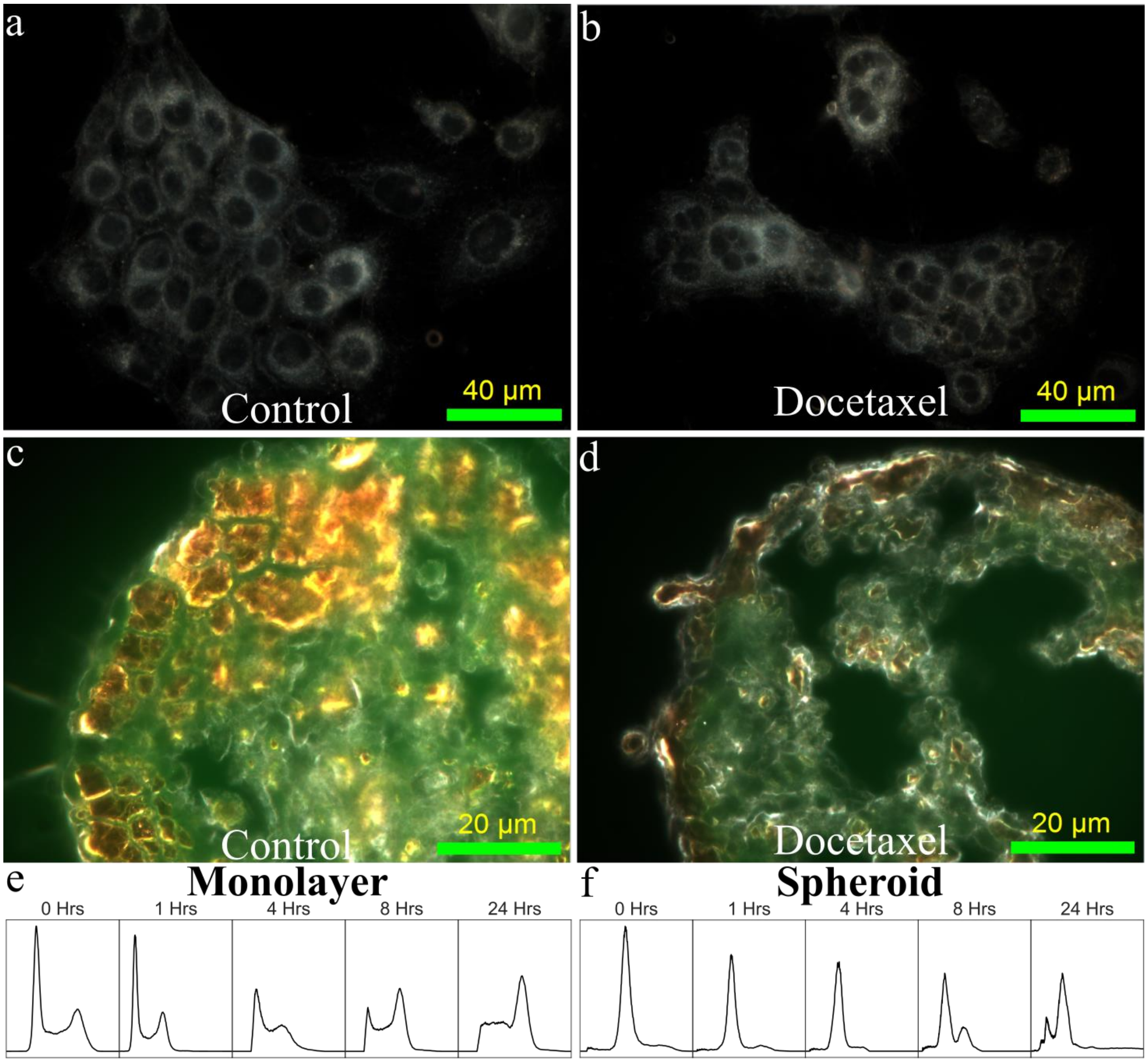

Figure S3. Effects of Docetaxel on CAL-27. (a, c) Darkfield images of a (a) monolayer of CAL-27 cells and a (c) CAL-27 spheroid section. (b, d) Darkfield images of a (b) monolayer of CAL-27 cells and a (d) CAL-27 spheroid section treated with docetaxel. Scale bar is 40 µm. (e-f) Cell cycle distribution after 0, 1, 4, 8, and 24 hours in a (e) monolayer of CAL-27 and a (f) CAL-27 spheroid.

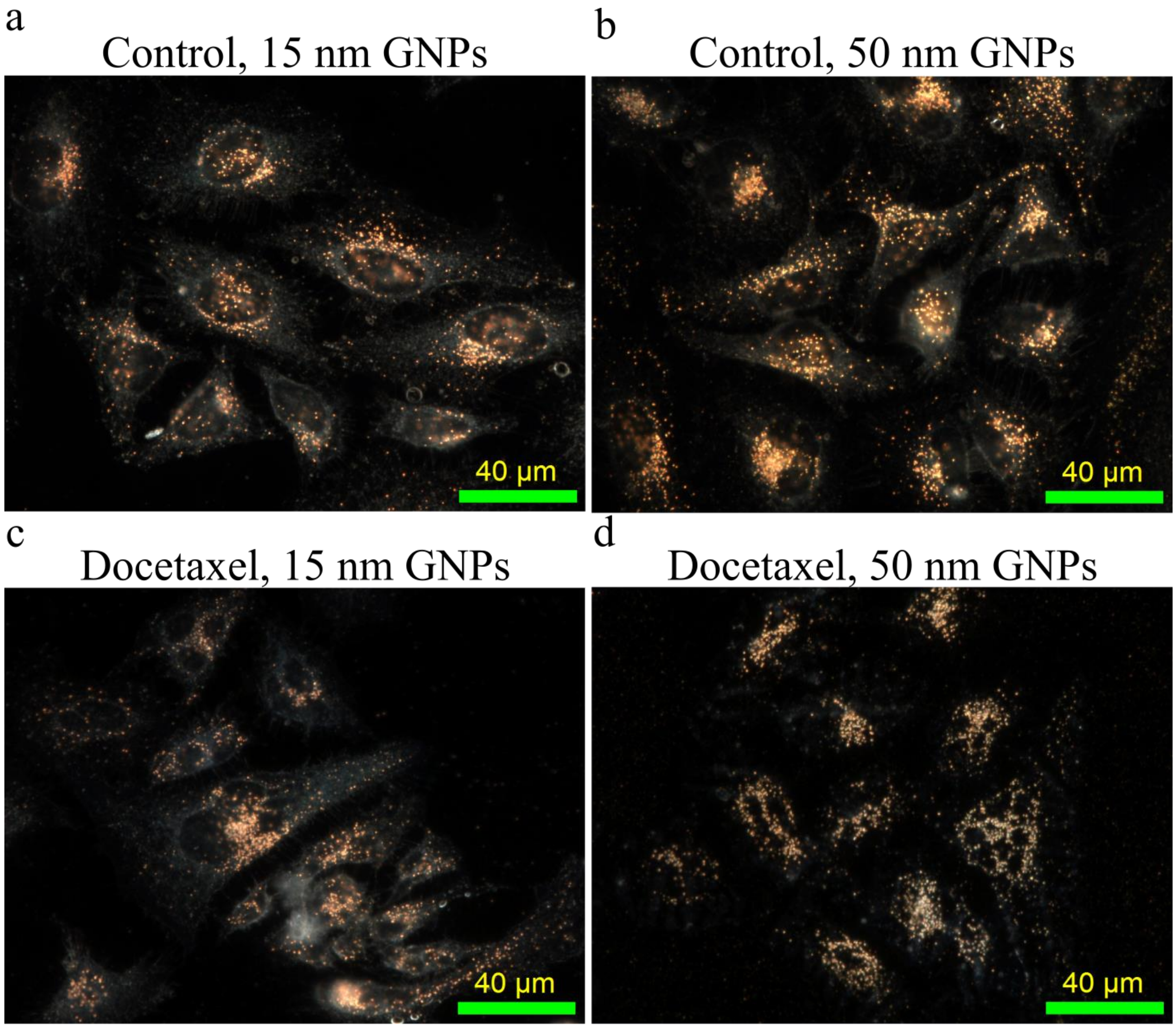

Figure S4. Darkfield images of HeLa in monolayer. (a-b) Darkfield imaging of HeLa cells with 15 nm and 50 nm functionalized gold nanoparticles without docetaxel. (c-d) Darkfield imaging of HeLa cells with 15 nm and 50 nm functionalized gold nanoparticles without docetaxel. Scale bar is 40 µm.

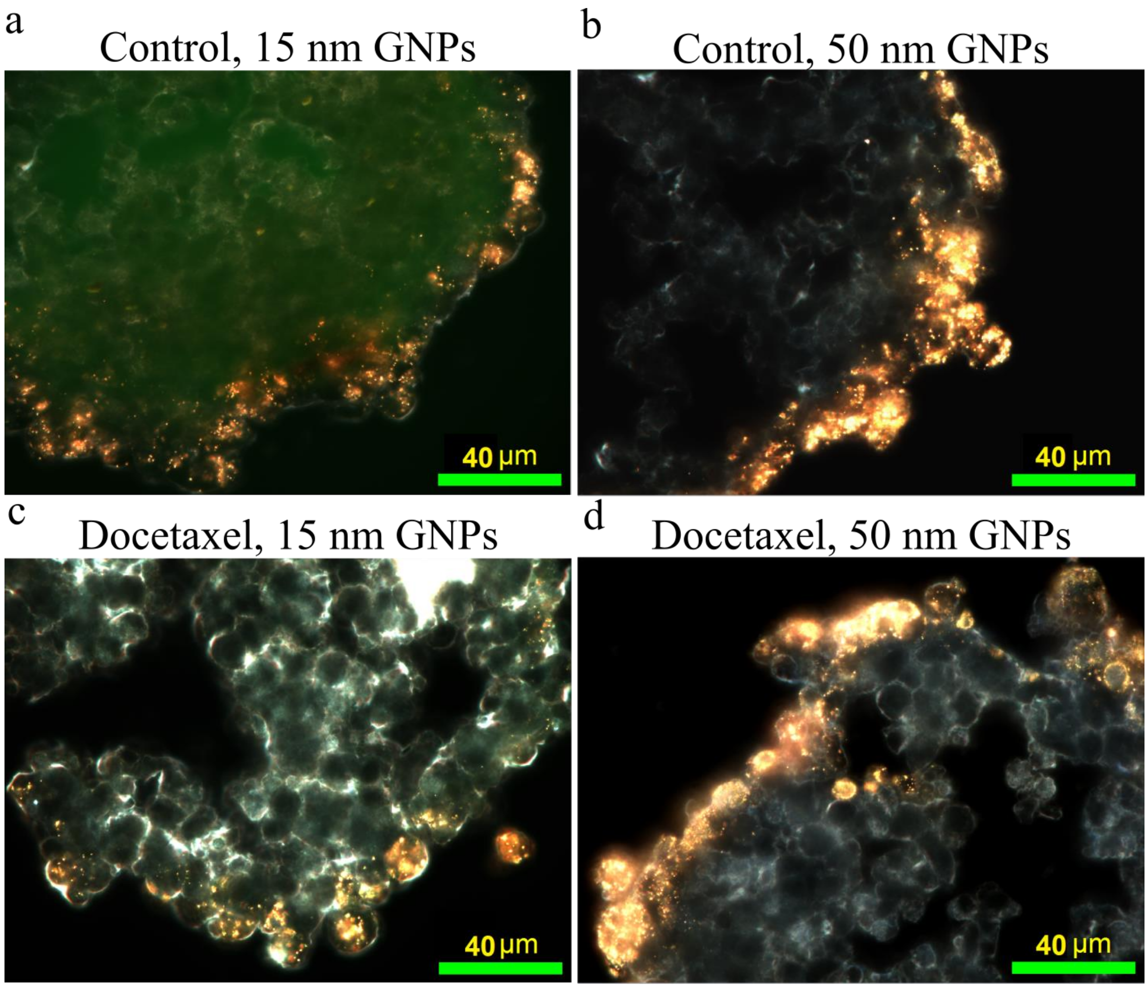

Figure S5. Darkfield images of HeLa in spheroids. (a-b) Darkfield imaging of HeLa spheroid with 15 nm and 50 nm functionalized gold nanoparticles without docetaxel. (c-d) Darkfield imaging of HeLa spheroid with 15 nm and 50 nm functionalized gold nanoparticles with docetaxel. Scale bar is 40 μm.

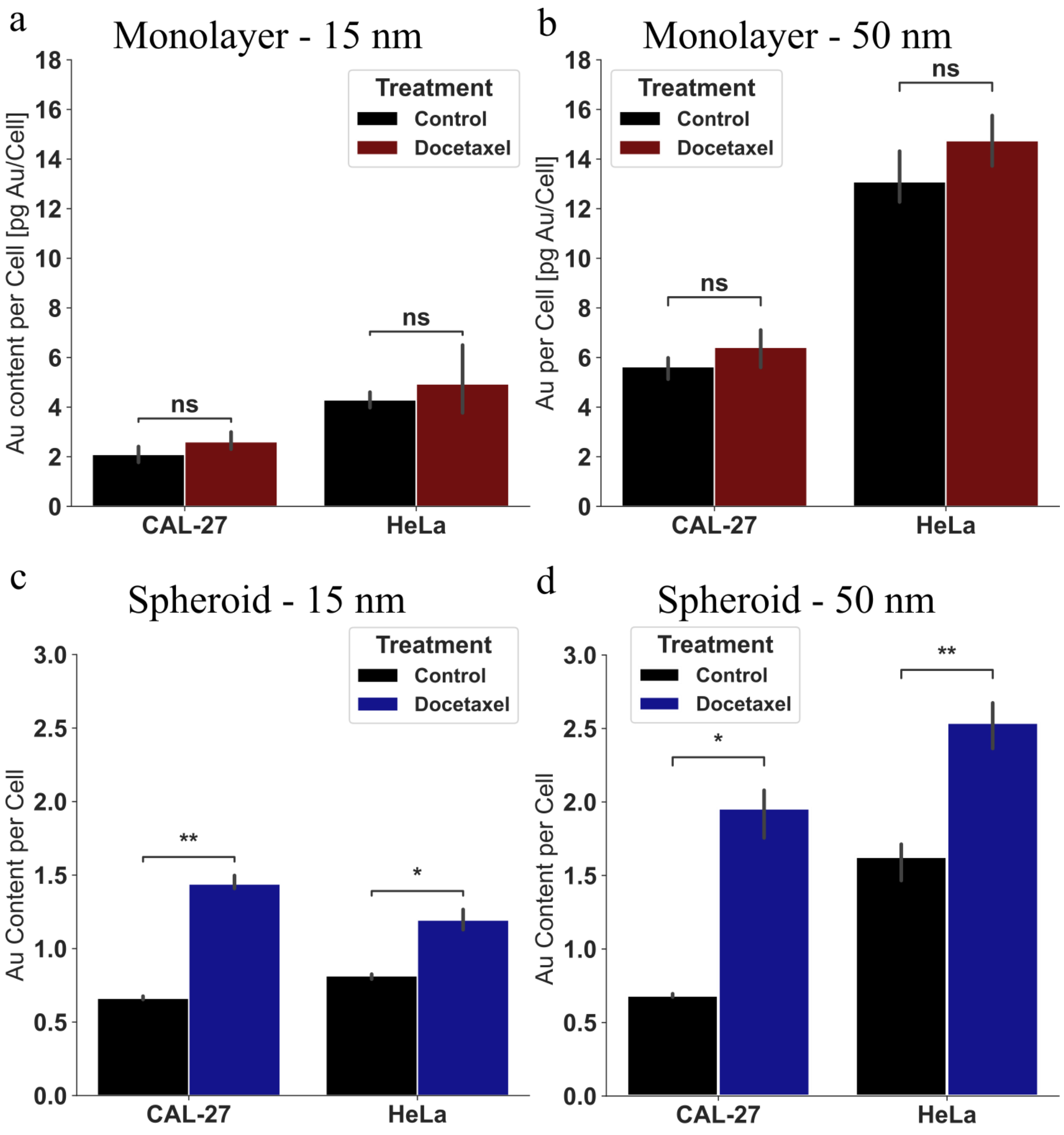

Figure S6. Gold content in monolayer and spheroids. (a-b) Total gold content due to uptake of the 15 nm and the 50 nm functionalized gold nanoparticles in monolayer, with and without docetaxel. (c-d) Total gold content due to the uptake of the 15 nm and the 50 nm functionalized gold nanoparticles in spheroids, with and without docetaxel. Error bars signify one standard deviation from average of three measurements. * indicates $0.01 < p < 0.05$, ** indicates $0.001 < p < 0.01$.
